# Rapid Authentication of Flowers of *Panax ginseng* and *Panax notoginseng* Using High-Resolution Melting (HRM) Analysis

**DOI:** 10.3390/molecules31030441

**Published:** 2026-01-27

**Authors:** Menghu Wang, Wenpei Li, Yafeng Zuo, Qianqian Jiang, Jincai Li, Wenhai Zhang, Xiangsong Meng

**Affiliations:** 1 College of Traditional Chinese Medicine, Bozhou University, Bozhou 236800, China; shapuofeng@163.com (M.W.); zuoyafeng@bzuu.edu.cn (Y.Z.); jiangqq_katherine@163.com (Q.J.); ahbanli@bzuu.edu.cn (J.L.); 2 Bozhou Communications Investment Group Co., Ltd., Bozhou 236800, China; liwenpei0208@dingtalk.com; 3 State Key Laboratory of Chemistry and Utilization of Carbon Based Energy Resources, College of Chemistry, Xinjiang University, Urumqi 830017, China

**Keywords:** high-resolution melting (HRM), DNA barcoding, *Panax ginseng* flower, *Panax notoginseng* flower, adulteration, *ITS2*, rapid identification

## Abstract

The flowers of *Panax ginseng* C. A. Mey. (PG) and *Panax notoginseng* (Burkill) F. H. Chen ex C. H. Chow (PN) are morphologically indistinguishable after drying, leading to prevalent adulteration that compromises product quality and consumer safety. To address this issue, we developed a rapid, closed-tube molecular authentication method based on high-resolution melting (HRM) analysis. Species-specific primer pairs were designed to target the conserved *ITS* and *rbcL-accD* regions, with PNG-2 selected as the optimal candidate owing to its stable genotyping performance and moderate GC content. Our results established GC content, rather than amplicon length, as the primary determinant of the melting temperature (*Tm*). Notably, the experimentally measured *Tm* values were consistently 0.7–1.5 °C higher than theoretical predictions, a discrepancy attributable to the stabilizing effect of the saturated fluorescent dye. To ensure maximum diagnostic reliability, the HRM results were cross-validated through a three-tier system comprising *ITS2* phylogenetic analysis, agarose gel electrophoresis, and Sanger sequencing. The practical utility and matrix robustness of the assay were further verified using a diversified validation cohort of 30 commercial samples, including 24 floral batches and 6 root-derived products (root slices and ultramicro powders). The HRM profiles demonstrated 100% concordance with DNA barcoding results, effectively identifying mislabeled products across different botanical matrices and processing forms. This methodology, which can be completed within 3 h, provides a significantly more cost-effective and rapid alternative to traditional sequencing-based methods for large-scale market surveillance and industrial quality control.

## 1. Introduction

Natural plant resources form the material foundation of traditional medicine and modern functional food industries [1,2,3,4]. Driven by global demand for “natural” and “healthy” products, the flowers of *Panax ginseng* (PG) and *Panax notoginseng* (PN) have become high-value ingredients due to their bioactive compounds, which exhibit significant antioxidant, anti-inflammatory, and immunomodulatory activities [5,6,7,8]. However, botanical materials often undergo morphological degradation after drying, making them prone to misidentification or intentional substitution during distribution [9]. PG and PN share extremely similar floral structures, posing identification challenges for non-specialists [10]. The substantial price difference between the two has led to frequent adulteration, creating an urgent need for rapid, reliable discrimination methods to ensure quality and consumer safety.

Conventional authentication methods, such as morphological observation and physicochemical analyses (e.g., TLC, HPLC-MS), are often limited by subjectivity, time-consuming preparation, and the chemical similarities inherent in closely related species [11,12,13,14,15]. Consequently, molecular biology advances—particularly DNA barcoding (e.g., *ITS2*)—have been widely established as objective benchmarks for accurate plant authentication [16,17,18,19,20,21,22]. Furthermore, various qPCR-based diagnostic platforms, including duplex real-time PCR and TaqMan probe assays, have demonstrated high sensitivity in identifying Panax species in roots and processed products [23,24]. While these hydrolysis probe methods offer exceptional specificity, their high costs and technical complexity can restrict routine industrial application [25].

High-resolution melting (HRM) analysis, an emerging “closed-tube” technique, monitors DNA denaturation behavior to enable rapid discrimination via melting curves, offering simplicity, sensitivity, and low risk of contamination [26,27,28,29,30,31]. Although Bar-HRM has been previously explored for identifying certain Panax species, existing studies focus predominantly on root materials and lack a systematic analysis of the relationships between amplification product characteristics and HRM stability, hindering methodological standardization [32,33].

This study describes the development and comprehensive validation of a rapid HRM-based assay for the discrimination of *Panax ginseng* (PG) and *Panax notoginseng* (PN) flowers. To satisfy the high standards of diagnostic reliability required for market supervision, our methodology was cross-validated through three analytical dimensions: *ITS2* phylogenetic analysis, agarose gel electrophoresis, and Sanger sequencing. Specifically, *ITS2* sequences served as the taxonomic gold standard, while electrophoresis confirmed the high specificity of the target 162 bp amplicons, and Sanger sequencing definitively linked thermodynamic melting shifts to specific nucleotide variations. This study provides a high-throughput, cost-effective tool for quality control and a systematic paradigm for standardizing HRM assays in botanical authentication.

## 2. Results and Discussion

**Figure 1:**
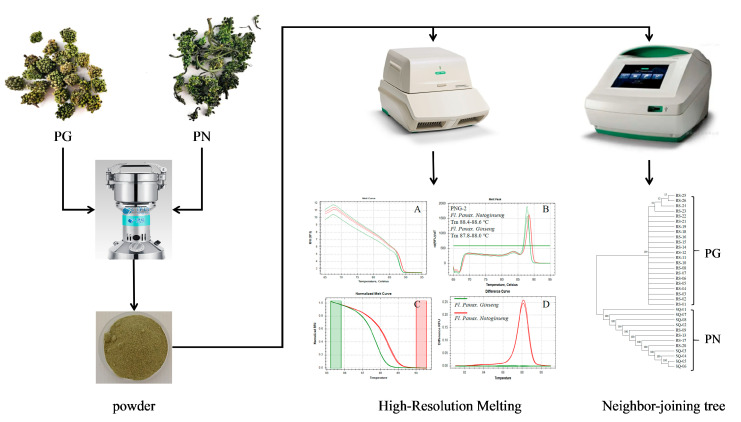
Schematic diagram of DNA extraction, HRM analysis, and DNA barcoding validation for flower samples of *P. ginseng* (PG) and *P. notoginseng* (PN). Note: (**A**) Melting curves, (**B**) melting peaks, (**C**) normalized curves. The green and red vertical lines demarcate the pre-melt and post-melt temperature regions, respectively, which were selected for fluorescence normalization to enable precise comparison of curve shapes between samples and (**D**) difference curves.

To provide a clear overview of the experimental design, the entire workflow for authenticating *P. ginseng* (PG) and *P. notoginseng* (PN) flowers is schematically illustrated in Figure 1. The process encompassed key stages from sample collection and DNA extraction through High-Resolution Melting (HRM) analysis, and was ultimately validated by DNA barcoding. The detailed results for each stage are presented below.

### 2.1. DNA Quality Assessment and Primer Screening

Genomic DNA was successfully extracted from all 34 plant samples, with concentrations exceeding 20 ng/μL and A260/A280 ratios ranging from 1.8 to 2.0. After dilution, all DNA templates were standardized to 20 ng/μL (Table S1). Sanger sequencing of the *ITS* and *rbcL-accD* regions was successfully completed for all samples, with sequencing results for PG and PN provided in Table S2.

**Figure 2:**
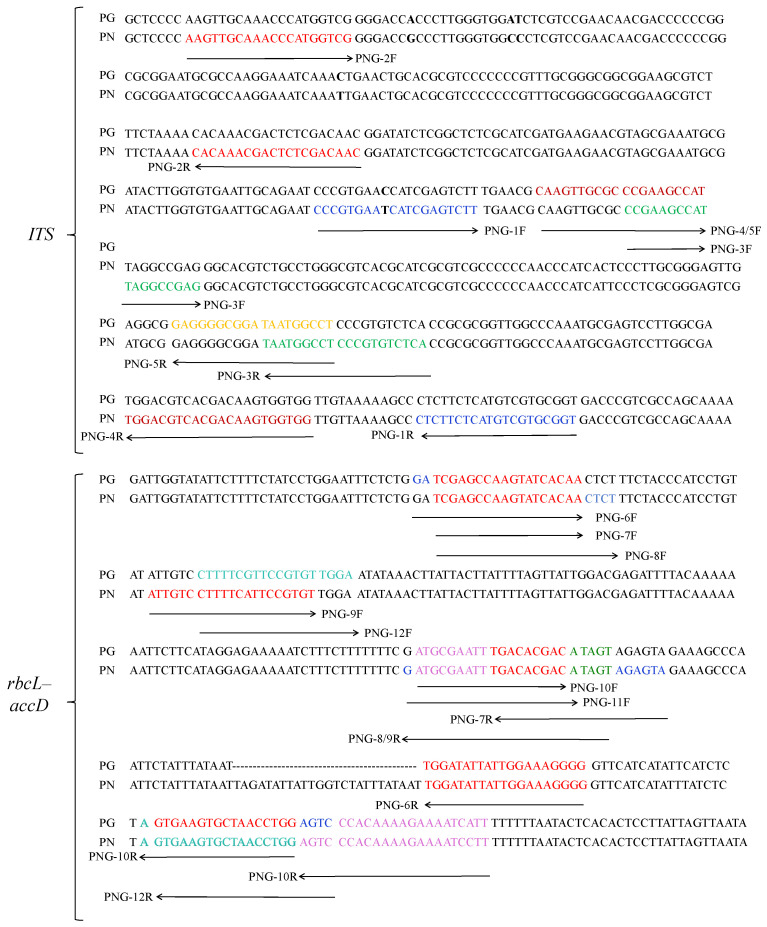
Primer design and sequence alignment for the target barcode regions. Note: The highlighted regions represent the genomic locations of the designed primers (PNG-1 to PNG-12). Different colors indicate sequence segments shared by multiple overlapping primer pairs, whereas single-colored regions denote primer-specific sequences flanking the species-discriminatory nucleotide variations.

Based on sequence alignments of the *ITS* and *rbcL-accD* regions between PG and PN, 12 primer pairs (designated PNG-1 to PNG-12) were designed, all of which amplified products containing species-specific nucleotide differences (Figure 2). Among these, five primer pairs targeted the *ITS* region and seven targeted the *rbcL-accD* region. Detailed information on primer pairs sequences, amplicon lengths, GC contents is summarized in Supplementary Table S3.

**Figure 3:**
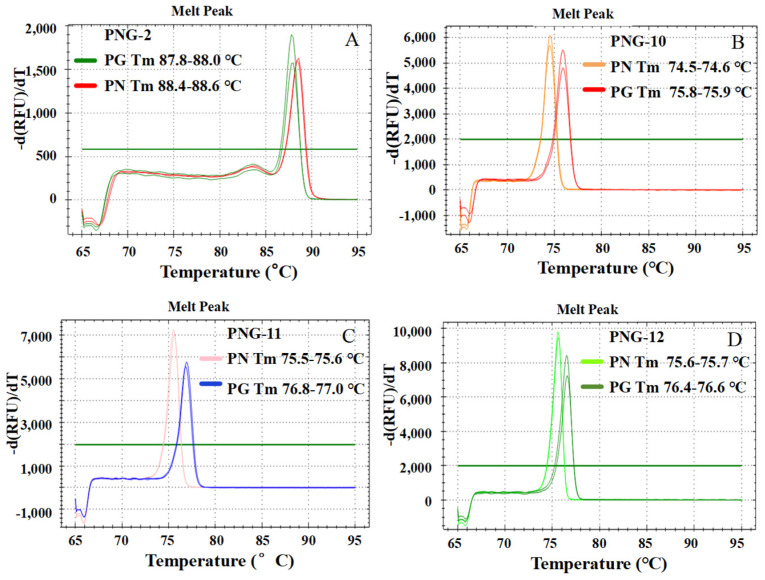
Melting peak profiles of PG (*P. ginseng* flowers) and PN (*P. notoginseng* flowers) amplified with primer pairs PNG-2 (**A**), PNG-10 (**B**), PNG-11 (**C**), and PNG-12 (**D**). Note: The peaks represent the negative derivative of fluorescence (-dF/dT) over temperature, with the maximum point of each peak corresponding to the melting temperature (Tm). The green horizontal line indicates the software-defined threshold for peak detection, ensuring that only signals above this baseline noise level are identified as valid melting events.

Melting curve analysis was conducted for production area samples using all 12 primer pairs, generating melting curves (Figure S1), melting peaks (Figure 3 and Figure S2), and corresponding melting temperatures (*Tm*). The *Tm* ranges are presented in Table S3. Results showed that seven primer pairs—PNG-2, PNG-6, PNG-7, PNG-8, PNG-10, PNG-11, and PNG-12—produced *Tm* differences > 0.3 °C between PG and PN (Table S3 and Figure 3 and Figure S2E–G), indicating potential for species discrimination. The remaining primer pairs exhibited no significant *Tm* differences and were ineffective for differentiation (Table S3 and Figure S2A–D,H). Further analysis revealed that while PNG-7 and PNG-8 could be distinguish between the two species, their *Tm* differences were small (<0.4 °C) (Figure S2F,G); additionally, their amplicons contained only single-nucleotide variants, resulting in limited discriminatory power due to the minimal impact on melting temperature (*Tm*) (Table S3). Thus, these two primer pairs were excluded from subsequent analyses.

### 2.2. HRM Analysis and Selection of the Optimal Primer

**Figure 4:**
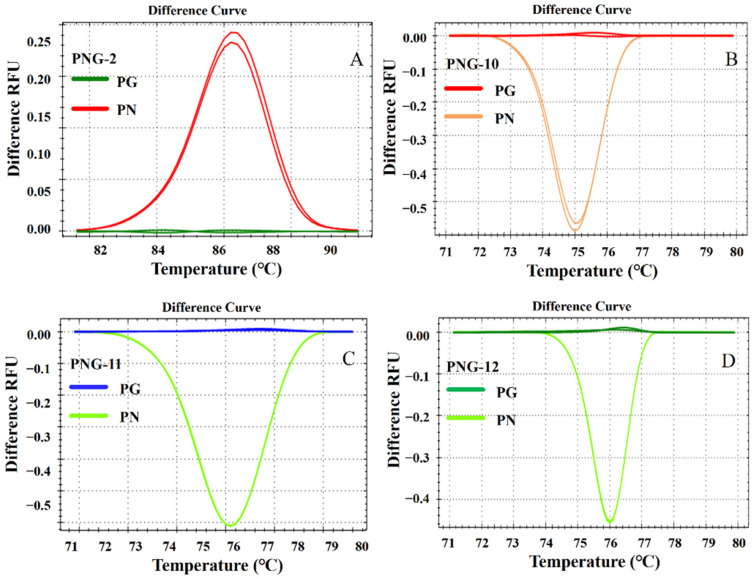
Difference curves of PG (*P. ginseng* flowers) and PN (*P. notoginseng* flowers) amplified with primer pairs PNG-2 (**A**), PNG-10 (**B**), PNG-11 (**C**), and PNG-12 (**D**).

HRM analysis was performed on the amplification products of the five candidate primer pairs (PNG-2, PNG-6, PNG-10, PNG-11, and PNG-12), generating distinct normalized melting curves (Figure S3) and difference curves (Figure 4 and Figure S4). All five pairs yielded clearly separated clusters with confidence levels exceeding 98%, demonstrating their baseline capacity for accurate species discrimination. Compared to raw melting peaks, the normalized (Figures S1B,F,J–L and S3A–E) and difference curves (Figure 3A–D, Figure S2E, Figure 4A–D and Figure S4) visualizations significantly enhanced the detection of subtle sequence-dependent variations, thereby improving interpretation accuracy.

However, the discriminatory power and robustness varied significantly among these candidates. While PNG-10, PNG-11, and PNG-12 produced larger inter-species ∆*Tm* values (1.2–1.5 °C) and prominent separation in difference plots (Figure 4B–D), their amplicons were characterized by low GC content (29.6–36.7%) and relatively low melting temperatures (~75 °C). Such AT-rich regions may compromise assay robustness when analyzing complex matrices or operating under variable PCR thermal cycling conditions. Similarly, PNG-6 (GC% = 30.9–32.6%) showed acceptable discrimination (Figure S4) but exhibited reduced thermal stability. Furthermore, primer pairs targeting limited single-nucleotide variants (e.g., PNG-7 and PNG-8) produced broader peaks and less distinct clustering (Figure S5A,B), likely due to insufficient sequence context variation to drive strong thermodynamic shifts.

Among the evaluated candidates, PNG-2 was identified as the optimal primer pair due to its superior balance of specificity, reproducibility, and thermodynamic stability. Although its inter-species ∆*Tm* is modest (0.6 °C), PNG-2 generated sharp, symmetric melting peaks at a high *Tm* (~88 °C) and provided unambiguous separation across all replicates (Figure 3A, Figure 4A and Figure S3A). Notably, PNG-2 targets a multi-polymorphic region rather than a single SNP, yielding more consistent and reliable signals. Its amplicon length (162 bp) and GC content (59.3–61.7%) align perfectly within the empirically determined optimal range for HRM (100–200 bp, 40–60% GC), ensuring high fluorescence intensity and minimal sensitivity to minor thermal fluctuations—critical advantages for the routine authentication of commercial samples.

### 2.3. Melting Temperature (Tm) Prediction and Experimental Validation

To evaluate the thermodynamic rationality of the HRM results, theoretical *Tm* values were predicted using the nearest-neighbor thermodynamic model, and DNA secondary structures were assessed via mfold (Table S3). The experimentally measured *Tm* values were consistently 0.7–1.5 °C higher than the theoretical predictions. This systematic positive deviation is primarily attributed to the stabilizing effects of the saturated intercalating dyes used in the HRM supermix [34,35], as well as the optimized cation concentrations (Mg^2+^, K^+^) which enhance duplex stability [36,37].

For the optimal primer pair PNG-2, the high GC content (approx. 60%) resulted in a predicted *Tm* of approximately 87 °C, which aligned closely with the experimentally observed peaks. In contrast, amplicons from PNG-6, PNG-10, PNG-11, and PNG-12 featured AT-rich regions, leading to lower overall GC contents (29–37%) and consequently reduced *Tm* values (74–76 °C). These results establish GC content, rather than absolute amplicon length, as the primary determinant of *Tm* shifts in this system.

Furthermore, mfold analysis predicted a minimal secondary structure (hairpin) in the *P. notoginseng* (PN) product of PNG-2 (∆G = −2.33 kcal/mol), while all other amplicons exhibited ∆G values ≥ −0.07 kcal/mol. The absence of significant secondary structures across all tested primer pairs resulted in single, highly symmetric melting peaks. This strong agreement between empirical measurements and thermodynamic predictions confirms the reliability and predictive accuracy of the established HRM assay for species discrimination.

### 2.4. Validation with Market Samples and Assessment of HRM Accuracy

**Figure 5:**
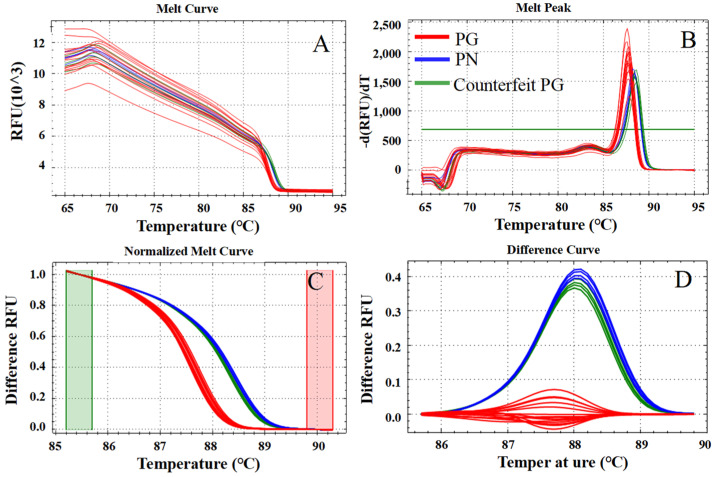
Rapid HRM screening results of samples purchased from markets and online platforms. Note: (**A**) Melting curves, (**B**) melting peaks, (**C**) normalized curves. The green and red vertical lines demarcate the pre-melt and post-melt temperature regions, respectively, which were selected for fluorescence normalization to enable precise comparison of curve shapes between samples, and (**D**) difference curves. Analysis was performed using Precision Melt Analysis™ software (v3.1, Bio-Rad Laboratories, Hercules, CA, USA).

To evaluate the practical utility and diagnostic accuracy of the established HRM assay, the optimal primer pair PNG-2 was applied to a comprehensive validation cohort of 30 commercial samples, including floral tissues, root slices, and ultramicro powders (Figure 5 and Figure S6). Among the 24 floral batches analyzed, HRM profiles revealed that four samples labeled as “PG” exhibited melting behaviors identical to the PN reference group (Figure 5A,C). Specifically, their *Tm* values shifted into the PN range (Figure 5B), and their difference curves clustered with the PN group with confidence levels exceeding 98% (Figure 5D), indicating significant market substitution or adulteration.

Furthermore, the assay demonstrated exceptional diagnostic robustness across different botanical matrices and processing forms. In tests involving two batches of PG root slices, two batches of PG ultramicro powder, and two batches of PN root slices, the *Tm* values for all PG root-derived products consistently ranged from 87.8 to 88.0 °C, while those for PN ranged from 88.4 to 88.6 °C (Figure S6). These profiles were perfectly congruent with the results obtained from floral tissues, confirming that the established HRM method can accurately authenticate commercial Panax products regardless of the medicinal part (root vs. flower) or physical state (slices vs. ultramicro powder). This high matrix stability underscores the method’s reliability for high-throughput quality control and forensic supervision within the natural plant supply chain.

**Figure 6:**
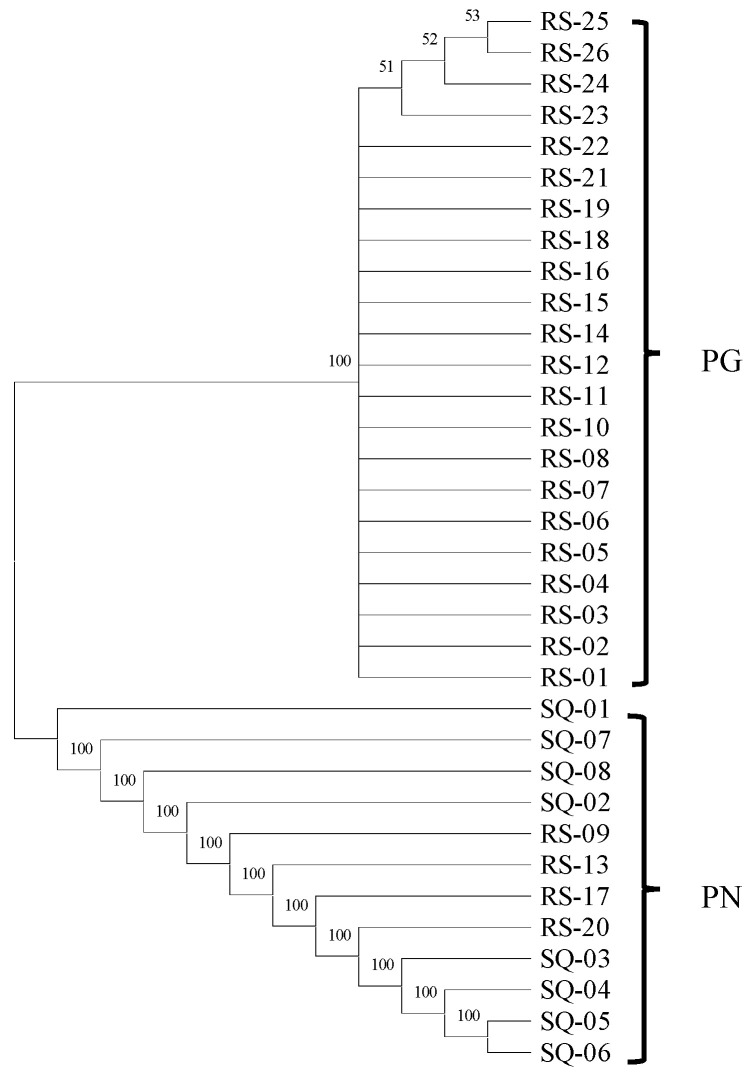
NJ tree of *ITS2* sequences discriminating PG (*P. ginseng* flowers) and PN (*P. notoginseng* flowers).

To verify these results, an NJ phylogenetic tree was constructed using *ITS2* sequences from all samples and GenBank references (Figure 6). The phylogenetic analysis showed that *P. ginseng* (PG) and *P. notoginseng* (PN) formed distinct, well-supported monophyletic clades with bootstrap support >95%. Crucially, the four samples identified as PN by HRM clustered precisely within the authentic PN clade, demonstrating 100% concordance between HRM-based screening and DNA barcoding identification. This cross-validation rigorously confirms the high accuracy and reliability of the established HRM method.

Furthermore, the molecular integrity of the HRM assay was corroborated by terminal verification of the PNG-2 amplicons. Agarose gel electrophoresis (Figure S7) revealed a single, sharp, and consistent band at 162 bp for all tested PG (*n* = 22) and PN (*n* = 12) samples, with no detectable non-specific amplification or primer-dimers. Subsequent Sanger sequencing and sequence alignment (Table S5) demonstrated that the amplicons were in 100% agreement with the theoretical target designs. The identification of conserved single nucleotide polymorphisms (SNPs) within the 162 bp region definitively validates that the distinct melting profiles observed are a direct consequence of specific nucleotide variations. Together, these results reinforce the HRM-based framework as a robust and precise tool for the molecular authentication of Panax floral products.

### 2.5. Discussion on HRM Advantages and Influencing Factors

The primary advantage of High-Resolution Melting (HRM) technology lies in its exceptional sensitivity to minor sequence variations, enabling the detection of single nucleotide polymorphisms (SNPs) and small insertions/deletions (InDels) via subtle shifts in melting profiles. In this study, five primer pairs were systematically screened, yielding amplicons (109–246 bp) with GC contents of 29.6–61.7% and inter-species ∆*Tm* values of 0.4–1.5 °C (Table S3). PNG-2 was identified as the optimal assay due to its ideal amplicon length (162 bp), moderate GC content (59.3–61.7%), and highly reproducible separation in normalized and difference plots (Figures S3A and 4A).

Our findings underscore the importance of polymorphism types in assay design. Primer pairs targeting single-nucleotide variants near amplicon termini (e.g., PNG-7 and PNG-8) generated minimal *Tm* shifts (<0.6 °C), leading to marginal cluster separation (Figure 4A and Figure S5A,B). In contrast, PNG-2 targets a multi-polymorphic region, producing larger and more robust thermodynamic differences [38,39].

Further analysis confirmed that GC content, rather than absolute amplicon length, is the primary determinant of *Tm*. For instance, PN exhibited lower *Tm* values than PG due to an AT-rich insertion that reduced its overall GC ratio (Figure 2). Notably, a systematic deviation was observed where experimental *Tm* values were 0.7–1.5 °C higher than theoretical predictions (Table S3). This is attributed to the stabilizing effect of saturated fluorescent dyes and cation concentrations (Mg^2+^) on double-stranded DNA [36,37].

The combined use of normalized and difference curves facilitates reliable discrimination even when ∆*Tm* is below 0.5 °C. When applied to 30 commercial samples, this integrated analytical approach successfully identified four mislabeled batches (Figure 5, Figure 6 and Figure S6). These findings reveal prevalent adulteration in the Panax market, which may compromise efficacy and safety.

### 2.6. Method Limitations and Future Perspectives

Despite the high accuracy and practical advantages demonstrated, several limitations must be addressed to facilitate the broader adoption of this HRM-based assay. First, HRM performance is fundamentally dependent on sequence divergence within conserved genomic regions (Table S2). Consequently, designing highly specific primers for hybrids or closely related species with minimal genetic variation remains a technical challenge. Second, botanical matrices—particularly floral tissues—often contain complex PCR inhibitors like polysaccharides and polyphenols. These compounds can impair amplification efficiency and compromise the resolution of melting curves. Future refinement of DNA extraction protocols and the integration of internal control genes are essential to enhance assay reliability across diverse sample qualities.

While the current assay has been validated for floral tissues, root slices, and ultramicro powders (Table S1), its performance in more complex matrices such as concentrated extracts or multi-herb formulations remains to be fully characterized. Future research will focus on optimizing DNA recovery from high-polysaccharide/polyphenol extracts and developing multiplex HRM assays to quantify adulteration levels in mixed herbal products. Notably, this validation was conducted within a single laboratory setting. Although our internal data demonstrate exceptional consistency and high confidence levels, inter-laboratory reproducibility remains a critical prerequisite for standardized diagnostic deployment. Future multi-center ring trials involving diverse experimental configurations and qPCR platforms will be indispensable to evaluate the cross-platform transferability and robustness of the proposed framework.

Looking forward, the evolution toward multiplex HRM assays will enable the simultaneous detection of multiple adulterants in complex mixtures. Integrating this analytical paradigm with portable qPCR instruments could facilitate a transition toward Point-of-Care Testing (POCT), allowing for rapid, on-site field deployment in herbal markets. Moreover, the universal applicability of this standardized HRM workflow can be extended to other commonly substituted medicinal plants, such as *Chrysanthemum morifolium* and *Lonicera japonica*. Through systematic optimization and cross-laboratory standardization, HRM analysis has the potential to become an indispensable tool for ensuring authenticity and traceability throughout the global traditional medicine supply chain.

## 3. Materials and Methods

### 3.1. Plant Materials

A total of 34 plant samples were collected, comprising 22 batches of flowers, 2 batches of root slices, and 2 batches of ultramicro powder for *P. ginseng* (PG), and 6 batches of flowers and 2 batches of root slices for *P. notoginseng* (PN). Among the PG samples, 2 batches were sourced from the ginseng-producing area of Changbai County (CC), Jilin Province, China (specimen codes: RS-01–RS-02); 4 batches of flowers, 2 batches of root slices, and 2 batches of ultramicro powder were purchased from local markets (LM) (RS-03–RS-06, RS-23–RS-24, RS-25–RS-26); and 18 batches were acquired from online platforms (OP) (RS-07–RS-22). For the PN samples, 2 batches originated from the *P. notoginseng*-producing area of Wenshan Prefecture (WS), Yunnan Province, China (SQ-01–SQ-02), and 4 batches of flowers and 2 batches of root slices were obtained from markets (SQ-03–SQ-06, SQ-07–SQ-08). All samples were authenticated morphologically by Professor Xiangsong Meng, Director of the Traditional Chinese Medicine Department at Bozhou University. Voucher specimens have been deposited in the Herbarium of Bozhou University, and detailed sample information is provided in Table S1.

### 3.2. DNA Extraction and Sequencing

Approximately 30 mg of each sample, including dried flowers, root slices, and ultramicro powder, was pulverized using a fully automatic cryogenic grinder (Jingxin Industrial Development Co., Ltd., Shanghai, China) to ensure complete tissue disruption and material homogeneity. Genomic DNA was extracted with the Ezup Column Plant Genomic DNA Extraction Kit (Sangon Biotech Co., Ltd., Shanghai, China) following the manufacturer’s protocol. A microvolume nucleic acid-protein analyzer (Nano-600+, Shanghai Jiapeng Technology Co., Ltd., Shanghai, China) was used to determine DNA concentration and purity; only samples with an A_260_/_A280_ ratio of 1.8–2.0 were used for subsequent experiments. The extracted DNA was diluted to 20 ng/μL with ddH_2_O and stored at 4 °C until use.

For samples from production areas (RS-01–RS-02, SQ-01–SQ-02), the *ITS* and *rbcL-accD* regions were amplified using universal primers; for all other samples, only *ITS* primers were used for amplification. The PCR products were purified and sent to Sangon Biotech Co., Ltd. (Shanghai, China) for Sanger bidirectional sequencing.

### 3.3. Primer Design

To develop a high-resolution melting (HRM) assay, *ITS* and *rbcL-accD* sequences from authenticated PG and PN samples were aligned using MEGA software (version 12.0; Sudhir Kumar, Philadelphia, PA, USA). Species-specific nucleotide variants were identified, and primers were designed to flank these sites, ensuring amplification of key discriminatory regions in both species. The comprehensive in silico design parameters, constraints used in Oligo Primer Analysis Software (version 7; Molecular Biology Insights, Inc., Colorado Springs, CO, USA), and detailed specifications for the primer pair PNG-2F/R are summarized in Supplementary Materials Table S4.

### 3.4. Melting Curve and High-Resolution Melting (HRM) Analysis

HRM analysis was conducted using the Bio-Rad CFX Connect Real-Time PCR System (Bio-Rad Laboratories, Hercules, CA, USA), integrated with Precision Melt Analysis™ software (v3.1). To ensure thermal uniformity and optimal fluorescence sensitivity, the instrument was pre-calibrated with the Precision Melt Analysis Calibration Kit (Bio-Rad Laboratories, Hercules, CA, USA). All reactions were performed using the HRM Supermix (Bio-Rad Laboratories, Hercules, CA, USA).

The 10 μL reaction system comprised 5 μL HRM Supermix, 0.2 μL of each forward and reverse primer (final concentration of 0.2 μM), 0.5 μL of standardized template DNA (20 ng), and nuclease-free water (Sangon Biotech Co., Ltd., Shanghai, China). The thermal cycling protocol consisted of an initial denaturation at 94 °C for 2 min, followed by 40 cycles of denaturation at 94 °C for 10 s and annealing/extension at 60 °C for 30 s, with fluorescence acquisition at the end of each cycle.

Immediately following amplification, the HRM phase was initiated by ramping the temperature from 65 °C to 94 °C at a precision rate of 0.1 °C per 5 s. Continuous fluorescence data (>1000 data points) were collected to generate high-resolution melting profiles. Raw melting data were processed using Precision Melt Analysis™ software, employing baseline and temperature normalization to yield normalized melt curve. Difference curves were constructed using sequence-verified *P. ginseng* (PG) samples as the baseline reference. Species discrimination was rigorously determined by the synchronized analysis of melting temperature (*Tm*) peaks and thermodynamic curve morphologies, which served as the primary diagnostic criteria for the HRM-based identification framework.

### 3.5. Melting Temperature (Tm) Prediction

The theoretical *Tm* values of PCR amplicons were predicted using the nearest-neighbor thermodynamic model proposed by SantaLucia (1998) [40]. Parameters for calculation included total enthalpy change (ΔH), total entropy change (ΔS), DNA concentration (50 nM), and salt ion concentration ([Na^+^] = 50 mM, derived from the HRM master mix). To evaluate the influence of DNA secondary structures (e.g., hairpins, hybridization) on *Tm*, free energy changes were predicted using the mfold web server (version 4.6; Michael Zuker, Troy, NY, USA) [40].

(1) $$Tm=\frac{∆H}{∆S+R\cdot ln(C_{T})}-273.15+f\left\lfloor {Na}^{+}\right\rfloor$$

ΔH represents the total enthalpy change (kcal/mol), and ΔS the total entropy change (cal/mol·K), for duplex formation. R is the gas constant (1.987 cal/mol·K), *Tm* is the melting temperature in degrees Celsius (°C), and C is the total strand concentration (mol/L) of the DNA duplex. The sodium ion concentration term serves as an empirical correction factor to account for ionic strength effects on duplex stability.

### 3.6. Applicability Assessment

To evaluate the discriminatory power, repeatability, and methodological robustness of the developed framework, the optimized HRM assay was applied to a diversified validation cohort of 30 commercial samples. This cohort integrated 24 floral batches (20 PG and 4 PN) alongside 6 root-derived samples, comprising 2 batches of PG root slices, 2 batches of PG ultramicro powder, and 2 batches of PN root slices. This expanded assessment was designed to verify the generalizability of the species-specific *Tm* signatures across different botanical matrices and processing forms. Synchronized HRM analysis was performed to establish the universal applicability of the PNG-2 primer set for high-throughput quality control throughout the Panax supply chain.

### 3.7. Method Validation

To evaluate the diagnostic reliability of the HRM assay for discriminating *P. ginseng* (PG) from *P. notoginseng* (PN), a comprehensive validation strategy was implemented. First, the internal transcribed spacer 2 (*ITS2*) regions were characterized as the taxonomic reference standard. The *ITS2* sequences were extracted from the *ITS* sequencing data of all collected samples utilizing the *ITS2* database (http://its2.bioapps.biozentrum.uni-wuerzburg.de (accessed on 3 September 2025); Universität Würzburg, Würzburg, Germany) and supplemented with homologous sequences retrieved from GenBank. Molecular identification was performed by constructing a Neighbor-Joining (NJ) phylogenetic tree in MEGA software (version 12.0; Sudhir Kumar, Philadelphia, PA, USA), with nodal support evaluated through 1000 bootstrap replicates. The resulting phylogenetic clustering patterns were systematically compared with the HRM-based categorization to rigorously assess the sensitivity and specificity of the methodology.

To further corroborate the molecular basis of the HRM profiles, PCR amplicons generated by the optimal PNG-2 primer set underwent terminal verification. The products were resolved by 1.5% (*w*/*v*) agarose (Sangon Biotech Co., Ltd., Shanghai, China) gel electrophoresis in 1× TAE buffer (Sangon Biotech Co., Ltd., Shanghai, China) to confirm target specificity and fragment purity. Only samples exhibiting a single, distinct band of the expected size (162 bp) without non-specific products were selected for subsequent analysis. Representative amplicons were then purified and subjected to bi-directional Sanger sequencing (Sangon Biotech Co., Ltd., Shanghai, China). These sequences were aligned with the reference *ITS2* barcodes to identify specific single nucleotide polymorphisms (SNPs). This multi-dimensional cross-validation—linking thermodynamic melting behavior directly to underlying nucleotide variations—ensures that the HRM assay serves as a robust and reliable surrogate for definitive species authentication.

## 4. Conclusions

This study successfully established a robust, closed-tube molecular diagnostic framework for the rapid discrimination of *P. ginseng* (PG) and *P. notoginseng* (PN) flowers using High-Resolution Melting (HRM) analysis. To ensure maximum diagnostic reliability, the HRM assay was rigorously cross-validated through a three-tier system: *ITS2* phylogenetic analysis, agarose gel electrophoresis, and Sanger sequencing. This comprehensive validation strategy definitively linked the thermodynamic melting profiles to specific single nucleotide polymorphisms (SNPs) within the *ITS2* and *rbcL-accD* regions, confirming that the observed *Tm* shifts are a direct consequence of specific nucleotide variations.

Compared to traditional morphological and physicochemical authentication methods, which are often subjective and limited by the lack of distinct features in processed materials [41,42], our HRM-based approach offers a superior objective benchmark. While established molecular techniques like Sanger sequencing remain the “gold standard” [43,44,45], their high cost and long turnaround times (2–5 days) hinder their utility for high-throughput screening. In contrast, the proposed HRM method enables the completion of a full analytical batch within 2–3 h [46,47,48], offering a superior economic advantage over traditional sequencing for high-throughput screening (Table S6). Notably, the assay demonstrated exceptional matrix stability and universal applicability across a diversified validation cohort of 30 commercial samples, accurately authenticating not only floral tissues but also root slices and ultramicro powders.

Furthermore, as a closed-tube system, this method minimizes the risk of amplicon carry-over contamination, a common challenge in routine molecular diagnostics [49,50,51,52]. Our findings regarding the determinant role of GC content in *Tm* stability provide essential data for the future standardization of HRM assays across different laboratory settings. This study provides an efficient, economically viable, and scientifically validated technical solution for the authenticity verification of Panax floral products, contributing a reliable tool for industrial quality control and the forensic supervision of the traditional medicine supply chain.

## Data Availability

The original contributions presented in this study are included in the article/Supplementary Materials. Further inquiries can be directed to the corresponding authors.

## Supplementary Materials

The following supporting information can be downloaded at: https://www.mdpi.com/article/10.3390/molecules31030441/s1, Figure S1: Melting curve of flowers of *P. ginseng* (PG) and *P. notoginseng* (PN) using primers PNG-1 (A), PNG-2 (B), PNG-3 (C), PNG-4 (D), PNG-5 (E), PNG-6 (F), PNG-7 (G),PNG-8 (H), PNG-9 (I), PNG-10 (J), PNG-11 (K), and PNG-12 (L); Figure S2: Melting peak analysis (*Tm*) of flowers of *P. ginseng* (PG) and *P. notoginseng* (PN) using primers PNG-1 (A), PNG-3 (B), PNG-4 (C), PNG-5 (D), PNG-6 (E), PNG-7 (F), PNG-8 (G), and PNG-9 (H); Figure S3: Normalized melting curves analysis of flowers of *P. ginseng* (PG) and *P. notoginseng* (PN) using primers PNG-2 (A), PNG-6 (B), PNG-10 (C), PNG-11 (D), and PNG-12 (E); Figure S4: Difference curves analysis of flowers of *P. ginseng* (PG) and *P. notoginseng* (PN) using primers PNG-6; Figure S5: Difference curves analysis of flowers of *P. ginseng* (PG) and *P. notoginseng* (PN) using primers PNG-7 and PNG-8; Figure S6. Rapid HRM screening results of *P. ginseng* and *P. notoginseng* processed products across different matrices; Figure S7. Agarose gel electrophoresis (1.5%) of the PCR products amplified with primer PNG-2 for the entire validation cohort; Table S1: Summary of sample sources and identification results for flowers of *P. ginseng* (PG) and *P. notoginseng* (PN); Table S2: *ITS* and *rbcL-accD* sequences of flowers of *P. ginseng* (PG) and *P. notoginseng* (PN); Table S3: Primer pair design and performance evaluation of high-resolution melting (HRM) analysis for discrimination of flowers of *P. ginseng*(PG) and *P. notoginseng*(PN); Table S4. Detailed in silico design parameters and specifications for the selected HRM primer pair PNG-2F/R; Table S5. Sequence identity analysis of the PNG-2 amplicons from *P. ginseng* (PG) and *P. notoginseng* (PN); Table S6: Comparison of advantages and disadvantages between DNA barcoding and HRM analysis.

## Abbreviations

The following abbreviations are used in this manuscript:

HRM	High-resolution melting
PG	*Panax ginseng*
PN	*Panax notoginseng*
PNG	Primer name
