# Supplementary material for: Rapid Authentication of Flowers of Panax ginseng and Panax notoginseng Using High-Resolution Melting (HRM) Analysis
Source: Molecules. 2026 Jan 27;31(3):441. doi: 10.3390/molecules31030441 (PMC12898732; doi:10.3390/molecules31030441)
Supplement: Supplementary file 1 [file molecules-31-00441-s001.zip › molecules-4022409-supplementary.pdf]

Supplementary Materials

Contents – 18 pages containing 7 figures and 6 Tables.

Table S1. Summary of sample sources and identification results for flowers of *P. ginseng* (PG) and *P. notoginseng* (PN).

No.	Sample Name	Collection Location	Species Identification by Melting Peak (*Tm*)	Species Identification by Difference Curve	Species Identification by *ITS2*	DNA Concentration (ng/μL)	A260/A280 Ratio
RS-01	PG	CC	PG	PG	PG	432	1.85
RS-02	PG	CC	PG	PG	PG	284	1.96
SQ-01	PN	WS	PN	PN	PN	569	1.87
SQ-02	PN	WS	PN	PN	PN	443	1.93
RS-03	PG	LM	PG	PG	PG	58	1.82
RS-04	PG	LM	PG	PG	PG	64	1.93
RS-05	PG	LM	PG	PG	PG	89	1.94
RS-06	PG	LM	PG	PG	PG	76	2.00

RS-07	PG	OP	PG	PG	PG	23	1.86
RS-08	PG	OP	PG	PG	PG	48	1.94
RS-09	PG	OP	PN	PN	PN	64	1.99
RS-10	PG	OP	PG	PG	PG	92	1.95
RS-11	PG	OP	PG	PG	PG	78	1.83
RS-12	PG	OP	PG	PG	PG	69	1.99
RS-13	PG	OP	PN	PN	PN	23	1.81
RS-14	PG	OP	PG	PG	PG	36	1.87
RS-15	PG	OP	PG	PG	PG	45	1.82
RS-16	PG	OP	PG	PG	PG	22	1.86
RS-17	PG	OP	PN	PN	PN	78	1.90
RS-18	PG	OP	PG	PG	PG	46	1.83
RS-19	PG	OP	PG	PG	PG	57	1.91
RS-20	PG	OP	PN	PN	PN	38	1.99

Collection Location:

RS-21	PG	OP	PG	PG	PG	42	1.89
RS-22	PG	OP	PG	PG	PG	49	1.96
RS-23	PG	LM	PG	PG	PG	26	1.81
RS-24	PG	LM	PG	PG	PG	32	1.85
RS-25	PG	LM	PG	PG	PG	41	1.83
RS-26	PG	LM	PG	PG	PG	38	1.80
SQ-03	PN	LM	PN	PN	PN	104	1.83
SQ-04	PN	LM	PN	PN	PN	89	1.88
SQ-05	PN	LM	PN	PN	PN	78	1.81
SQ-06	PN	LM	PN	PN	PN	56	1.90
SQ-07	PN	LM	PN	PN	PN	36	1.83
SQ-08	PN	LM	PN	PN	PN	29	1.80

Abbreviations denote sample sources: CC, Changbai County, Jilin Province (a geographic locality for *P. ginseng*); WS, Wenshan Prefecture, Yunnan Province (a geographic locality for *P. notoginseng*); OP, online platforms; LM, local commercial markets.

Table S2. *ITS* and *rbcL-accD* sequences of flowers of *P. ginseng* (PG) and *P. notoginseng* (PN).

DNA barcoding region	sequenced nucleotide sequence
PG<br>ITS	GTCGAAACCTGCATAGCAGAACGACCCGCGAACACGTTACAATACCGGGTGAGGGACGAGGGGTGCGCAAGCTCCCCAAGTTGCAAACCCATGGTCGGGGACCACCCTTGGGTGGATCTCGTCC<br>GAACAACGACCCCCCGGCGCGGAATGCGCCAAGGAAATCAAACTGAACTGCACGCGTCCCCCCCGTTTGCGGGCGGCGGAAGCGTCTTTCTAAAACACAAACGACTCTCGACAACGGATATCTC<br>GGCTCTCGCATCGATGAAGAACGTAGCGAAATGCGATACTTGGTGTGAATTGCAGAATCCCGTGAACCATCGAGTCTTTGAACGCAAGTTGCGCCCGAAGCCATTAGGCCGAGGGCACGTCTGCCT<br>GGGCGTCACGCATCGCGTCGCCCCCCAACCCATCACTCCCTTGCGGGAGTTGAGGCGGAGGGGCGGATAATGGCCTCCCGTGTCTCACCGCGCGGTTGGCCCAAATGCGAGTCCTTGGCGATGGA<br>CGTCACGACAAGTGGTGGTTGTAAAAAGCCCTCTTCTCATGTCGTGCGGTGACCCGTCGCCAGCAAAAGCTCTCATGACCCTGTTGCGCCGTCCTCGACGTGCGCTCCGACCGCGACCCC
PN<br>ITS	GTCGAAACCTGCACAGCAGAACGACCCGCGAACAAGTTACAATACCGGGTGAGGGATGAGGGGTGCGTAGGCTCCCCAAGTTGCAAACCCATGGTCGGGGACCGCCCTTGGGTGGCCCTCGTCC<br>GAACAACGACCCCCCGGCGCGGAATGCGCCAAGGAAATCAAATTGAACTGCACGCGTCCCCCCCGTTTGCGGGCGGCGGAAGCGTCTTTCTAAAACACAAACGACTCTCGACAACGGATATCTCG<br>GCTCTCGCATCGATGAAGAACGTAGCGAAATGCGATACTTGGTGTGAATTGCAGAATCCCGTGAATCATCGAGTCTTTGAACGCAAGTTGCGCCCGAAGCCATTAGGCCGAGGGCACGTCTGCCTG<br>GGCGTCACGCATCGCGTCGCCCCCCAACCCATCATTCCCTCGCGGGAGTCGATGCGGAGGGGCGGATAATGGCCTCCCGTGTCTCACCGCGCGGTTGGCCCAAATGCGAGTCCTTGGCGATGGACG<br>TCACGACAAGTGGTGGTTGTTAAAAGCCCTCTTCTCATGTTGTGCGGTGACCCGTCGCCAGCAAAAGCTCTCATGACCCTGTTGCGCTGTCCTCGACGCGCGCTCCGACCGCGACCCC
PG rbcL-accD	AATAATTACCGTTCGTTCTCTTAATTGAATTGCAATTAAACTCGGCCCAATCTTTTACTAAAAGGATTGAGCCGAATACAAAGACCCTATTGCATATATATATTATATTTTTTAGATAGATACATACTTATC<br>TAGATATAGAAGAATATACAAAATCTAAGACTAAACAACTCAAACGTTTCTATTGGTGTGTTGAATCCACAATTAATCCGATGGATCCTTAGGATTGGTATATTCTTTTCTATCCTGGAATTTCTCTGGA<br>TCGAGCCAAGTATCACAACTCTTTCTACCCATCCTGTATATTGTCCTTTTCGTTCCGTGTTGGAATATAAACTTATTACTTATTTTAGTTATTGGACGAGATTTTACAAAAAAATTCTTCATAGGAGAAA<br>AATCTTTCTTTTTTTCGATGCGAATTTGACACGACATAGTAGAGTAGAAAGCCCAATTCTATTTATAATTGGATATTATTGGAAAGGGGGTTCATCATATTCATCTCTAGTGAAGTGCTAACCTGGAGTC<br>CCACAAAAGAAAATCATTTTTTTTAATACTCACACTCCTTATTAGTTAATAATCCTGGTGATTGGATTTCTATGTTTATTTTGACGGGAAAAAGAGATTCAAATAAAAGATTTTTCATCGA
PN rbcL-accD	AATAATTACCGTTCGTTCTCTTAATTGAATTGCAATTAAACTCGGCCCAATCTTTTACTAAAAGGATTGAGCCGAATACAAAGACCCTATTGCATATATATATTATATTTTTTAGATAGATACATACTTATC<br>TAGATATAGAAGAATATACAAAATCTAAGACTAAACAAGTCAAACGTTTCTATTGGTGTGTTGAATCCACAATTAATCCGATGGATCCTTAGGATTGGTATATTCTTTTCTATCCTGGAATTTCTCTGGA<br>TCGAGCCAAGTATCACAACTCTTTCTACCCATCCTGTATATTGTCCTTTTCATTCCGTGTTGGAATATAAACTTATTACTTATTTTAGTTATTGGACGAGATTTTACAAAAAAATTCTTCATAGGAGAAAA<br>ATCTTTCTTTTTTTCGATGCGAATTTGACACGACATAGTAGAGTAGAAAGCCCAATTCTATTTATAATTAGATATTATTGGTCTATTTATAATTGGATATTATTGGAAAGGGGGTTCATCATATTTATCTCT<br>AGTGAAGTGCTAACCTGGAGTCCCACAAAAGAAAATCCTTTTTTTTAATACTCACACTCCTTATTAGTTAATAATCCTGGTGATTGGATTTCTATGTTTATTTTGACGGGAAAAAGAGATTCAAATAAA

	AGATTTTCATCGA

Table S3. Primer pair design and performance evaluation of high-resolution melting (HRM) analysis for discrimination of flowers of *P. ginseng*(PG) and *P. notoginseng*(PN).

DNA barcoding region	Primer name	Primer sequence (5′–3′)	Amplicon characteristics	HRM analysis results	Predicted *Tm* value (℃)	Note
ITS2	PNG-1F<br>PNG-1R	CCCGTGAA**C**CATCGAGTCTT<br>ACCGCACGACATGAGAAGAG	243 bp; C/T mismatch at position 9; GC content: 62.1% (PG), 61.7% (PN)	Identical melting peak *Tm* (89.8 ℃); no separation in normalized or difference curves (confidence ≤80%)	–	High GC content but insufficient sequence divergence for effective discrimination
ITS2	PNG-2F<br>PNG-2R	AAGTTGCAAACCCATGGTCG<br>GTTGCCGAGAGTCGTTTGTG	162 bp; GC content: 59.3% (PG), 61.7% (PN); ΔGC% = 2.47%	*Tm* range: 87.8–88.0 ℃ (PG), 88.4–88.6 ℃ (PN) (Δ*Tm* = 0.4–0.8 ℃); clear differentiation in both melting and difference curves (confidence ≥95%)	86.5 (PG), 87.8 (PN)	High GC% dominates *Tm* elevation; weak secondary structure stability (ΔG = −0.46 to −2.33 kcal/mol)
ITS2	PNG-3F<br>PNG-3R	CCGAAGCCATTAGGCCGAG<br>TGAGACACGGGAGGCCATTA	119 bp; GC content: 67.2% for both species	*Tm*: 88.2 ℃ (PG), 88.2–88.4 ℃ (PN); not reliably distinguishable (confidence ≤80%)	–	Identical GC content and minimal structural differences result in low resolution
ITS2	PNG-4F	CAAGTTGCGCCCGAAGCCAT	185 bp; GC content: 66.0% for both species	*Tm* = 89.4 ℃ for both; no curve separation (confidence ≤80%)	–	Identical GC content and length lead to identical melting behavior

	PNG-4R	CCACCACTTGTCGTGACGTCCA				
ITS2	PNG-5F<br>PNG-5R	CAAGTTGCGCCCGAAGCCAT<br>AGGCCATTATCCGCCCCTC	118 bp; GC content: 66.9% for both species	*Tm* = 88.4 ℃ for both; not distinguishable (confidence ≤80%)	–	Identical GC content and length result in identical melting profiles
rbcl-accD	PNG-6F<br>PNG-6R	GATCGAGCCAAGTATCACAA<br>CCCCTTTCCAATAATATCCA	PG: 221 bp, PN: 246 bp (+25 bp insertion); GC content: 32.6% (PG), 30.9% (PN); ΔGC% = 1.69%	*Tm*: 77.0–77.1 ℃ (PG), 76.6–76.7 ℃ (PN) (Δ*Tm* = 0.4–0.5 ℃); distinguishable (confidence ≥95%)	76.2 (PG), 75.5 (PN)	AT-rich insertion in PN reduces GC%, partially counteracting the length-induced *Tm* increase
rbcl-accD	PNG-7F<br>PNG-7R	TCGAGCCAAGTATCACAA<br>TACTCTACTATGTCGTGTCA	177 bp; single C/A variation; GC content: 32.2% (PG), 31.6% (PN); ΔGC% = 0.56%	*Tm*: 76.4 ℃ (PG), 76.0–76.1 ℃ (PN) (Δ*Tm* = 0.3–0.4 ℃); distinguishable (confidence ≥95%)	75.7 (PG), 75.3 (PN)	No significant secondary structure (ΔG ≈ +0.01 to +0.84 kcal/mol); stable *Tm* difference
rbcl-accD	PNG-8F<br>PNG-8R	TCGAGCCAAGTATCACAACTCT<br>ACTATGTCGTGTCAAATTCGCATC	170 bp; single C/A variation; GC content: 32.4% (PG), 31.8% (PN); ΔGC% = 0.59%	*Tm*: 76.3–76.4℃ (PG), 75.9–76.0 ℃ (PN) (ΔTm = 0.3–0.5 ℃); distinguishable (confidence ≥95%)	75.7 (PG), 75.2 (PN)	Similar to PNG-7; primer extension does not significantly affect discrimination capability
rbcl-accD	PNG-9F<br>PNG-9R	ATTGTCCTTTTCGTTCCGTGT<br>ACTATGTCGTGTCAAATTCGCATC	131 bp; G/A mismatch at position 13; GC content: 29.0% (PG), 28.2% (PN)	*Tm*: 74.7–74.8 ℃ (PG), 74.6 ℃ (PN) (Δ*Tm* ≤ 0.2 ℃); not distinguishable (confidence ≤80%)	–	Insufficient *Tm* difference for reliable discrimination
rbcl-accD	PNG-10F<br>PNG-10R	ATGCGAATTTGACACGAC<br>CCAGGTTAGCACTTCACT	PG: 109 bp, PN: 134 bp (+25 bp insertion); GC content: 36.7% (PG), 32.1% (PN); ΔGC% = 4.61%	*Tm*: 75.8–75.9 ℃ (PG), 74.5–74.6 ℃ (PN) (Δ*Tm* = 1.2–1.4 ℃); clearly distinguishable (confidence ≥95%)	75.0 (PG), 73.8 (PN)	Short fragment lacks stable secondary structure; AT-rich insertion in PN significantly lowers *Tm*
rbcl-accD	PNG-11F	GATGCGAATTTGACACGACA	PG: 132 bp, PN: 157 bp (+25 bp insertion); GC content: 36.4% (PG), 33.1% (PN);	*Tm*: 76.8–77.0 ℃ (PG), 75.5–75.6 ℃ (PN) (Δ*Tm* = 1.2–1.5 ℃); clearly distinguishable	76.0 (PG), 74.7 (PN)	*Tm* influenced by both amplicon length and GC content

	PNG-11R	AATGATTTTCTTTTGTGGGACT	ΔGC% = 3.24%	(confidence ≥95%)		
rbcL-accD	PNG-12F<br>PNG-12R	CTTTTCGTTCCGTGTTGGA<br>GACTCCAGGTTAGCACTTCAC	PG: 215 bp, PN: 240 bp (+25 bp insertion); GC content: 32.1% (PG), 29.6% (PN); ΔGC% = 2.51%	*Tm*: 76.4–76.6 ℃ (PG), 75.6–75.7 ℃ (PN) (Δ*Tm* = 0.7–1.0 ℃); distinguishable (confidence ≥95%)	75.6 (PG), 74.8 (PN)	Predicted trend matches experimental results; slightly negative ΔG in PN does not affect overall melting behavior

Note: The term "DNA barcode region" refers to DNA barcode loci used in this study. ITS is the internal transcribed spacer (non-coding), rbcL-accD are chloroplast intergenic spacers.

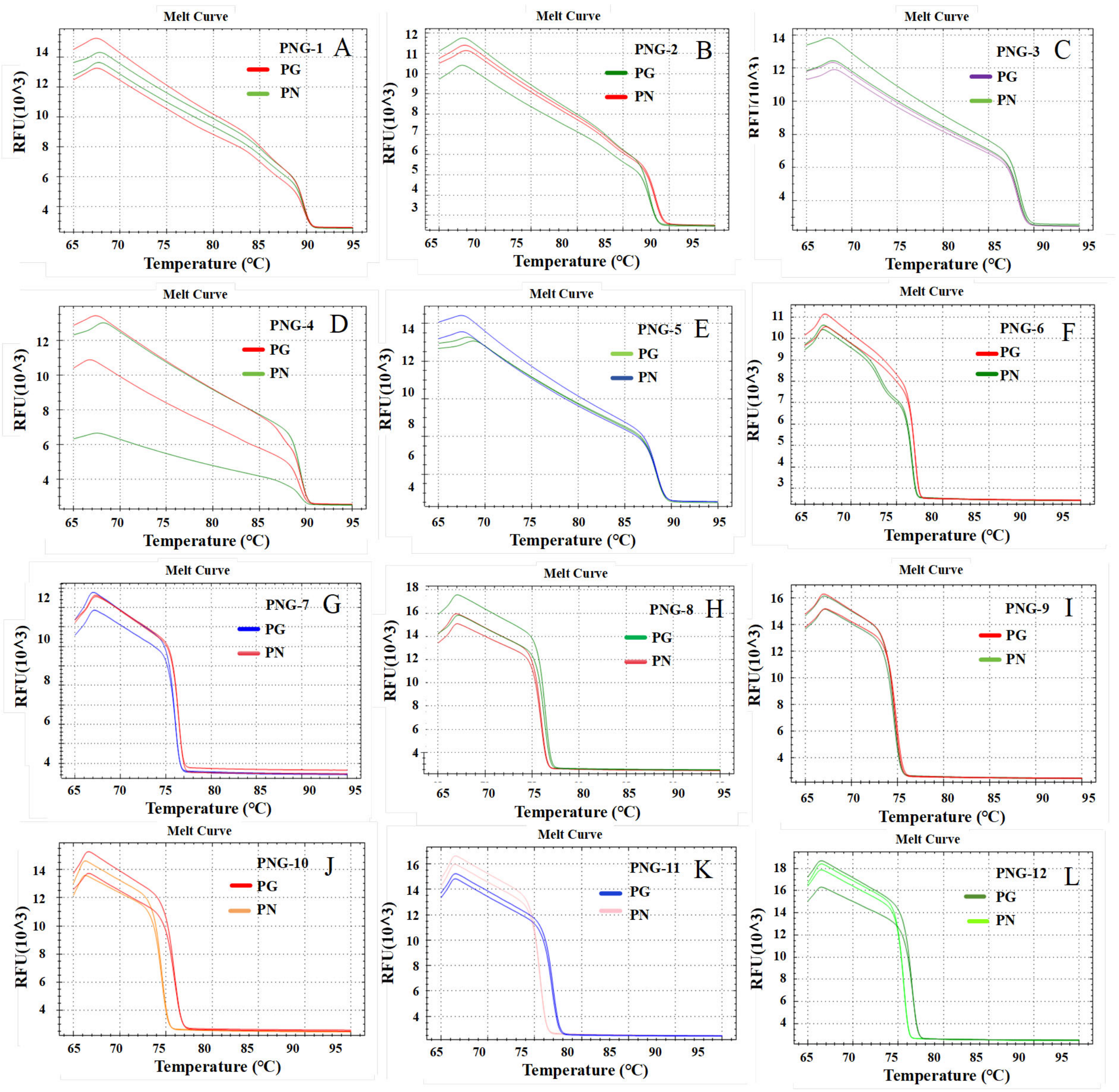

Figure S1. Melting curve of flowers of *P. ginseng* (PG) and *P. notoginseng* (PN) using primers PNG-1 (A), PNG-2 (B), PNG-3 (C), PNG-4 (D), PNG-5 (E), PNG-6 (F), PNG-7 (G) ,PNG-8 (H), PNG-9 (I), PNG-10 (J) , PNG-11 (K), and PNG-12 (L).

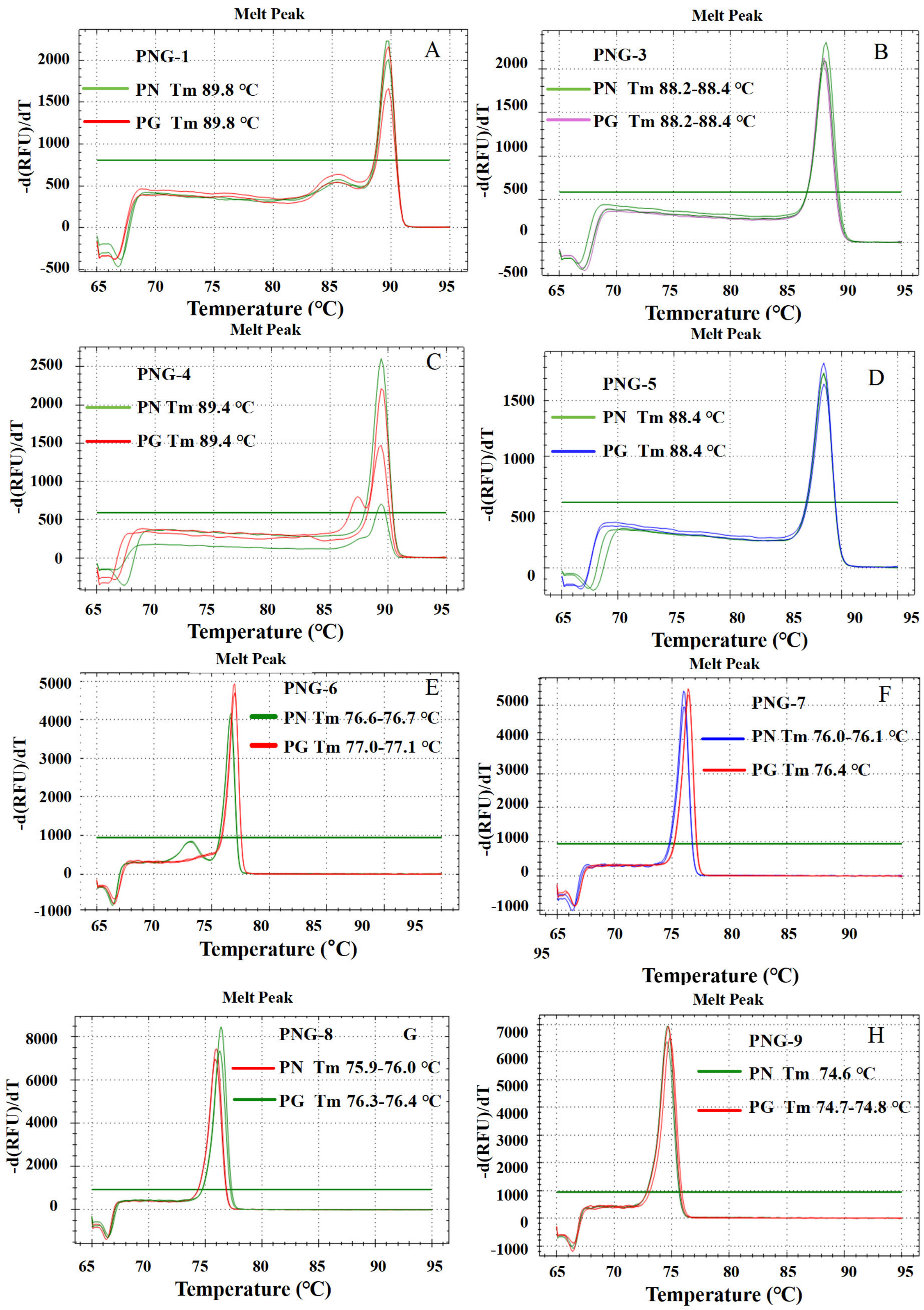

Figure S2. Melting peak analysis (*Tm*) of flowers of *P. ginseng* (PG) and *P. notoginseng* (PN) using primers PNG-1(A), PNG-3(B), PNG-4(C), PNG-5(D), PNG-6(E), PNG-7(F), PNG-8(G), and PNG-9(H).

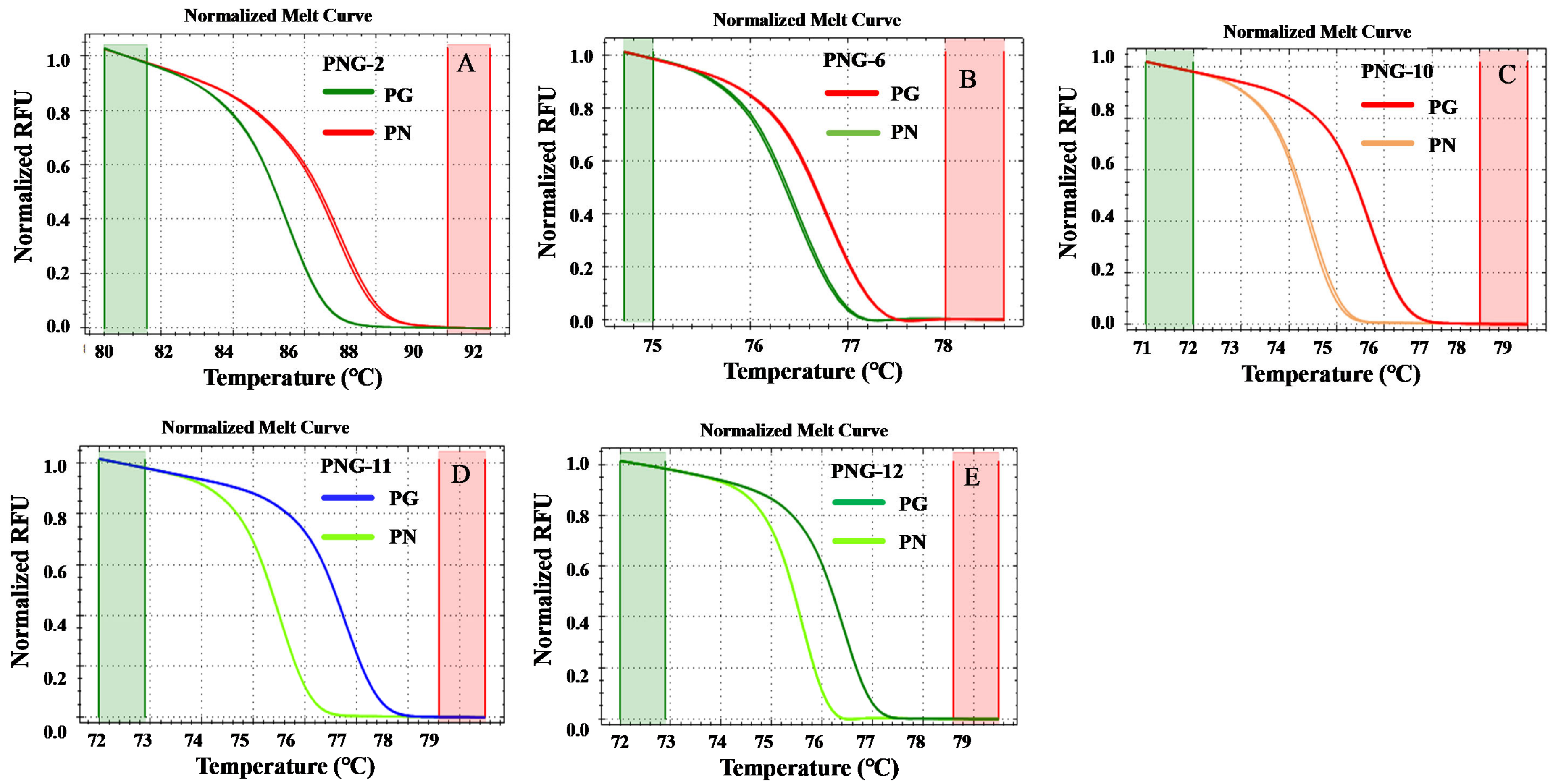

Figure S3. Normalized melting curves analysis of flowers of *P. ginseng* (PG) and *P. notoginseng* (PN) using primers PNG-2 (A), PNG-6 (B), PNG-10 (C), PNG-11 (D), and PNG-12 (E).

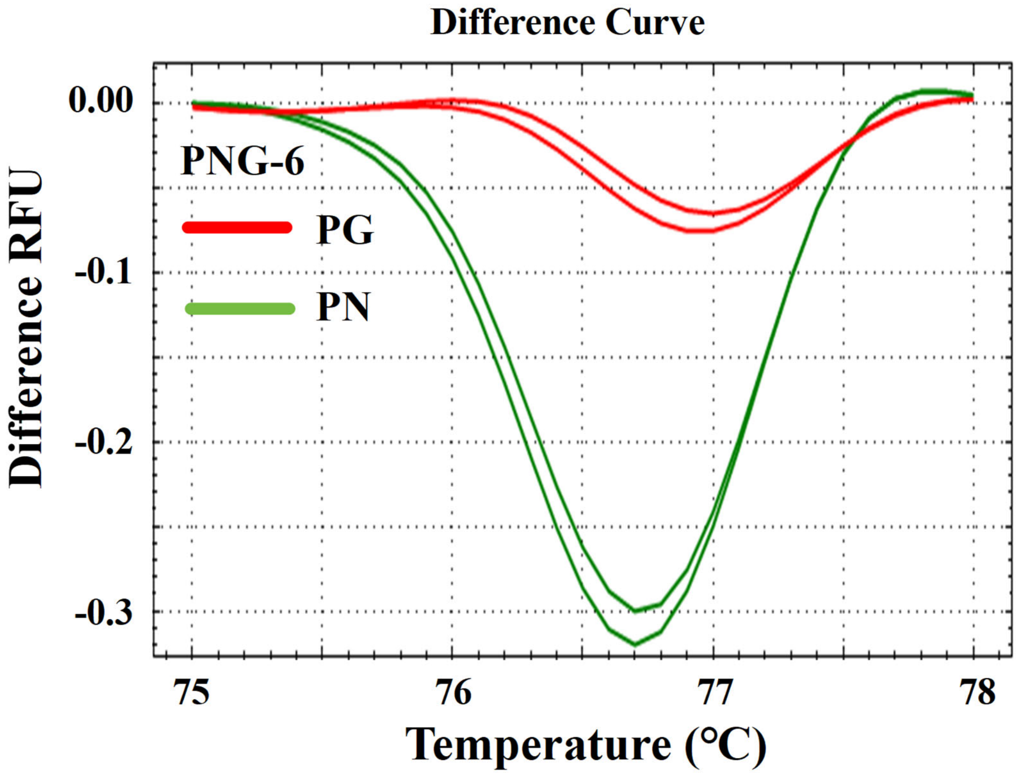

Figure S4. Difference curves analysis of flowers of *P. ginseng* (PG) and *P. notoginseng* (PN) using primers PNG-6.

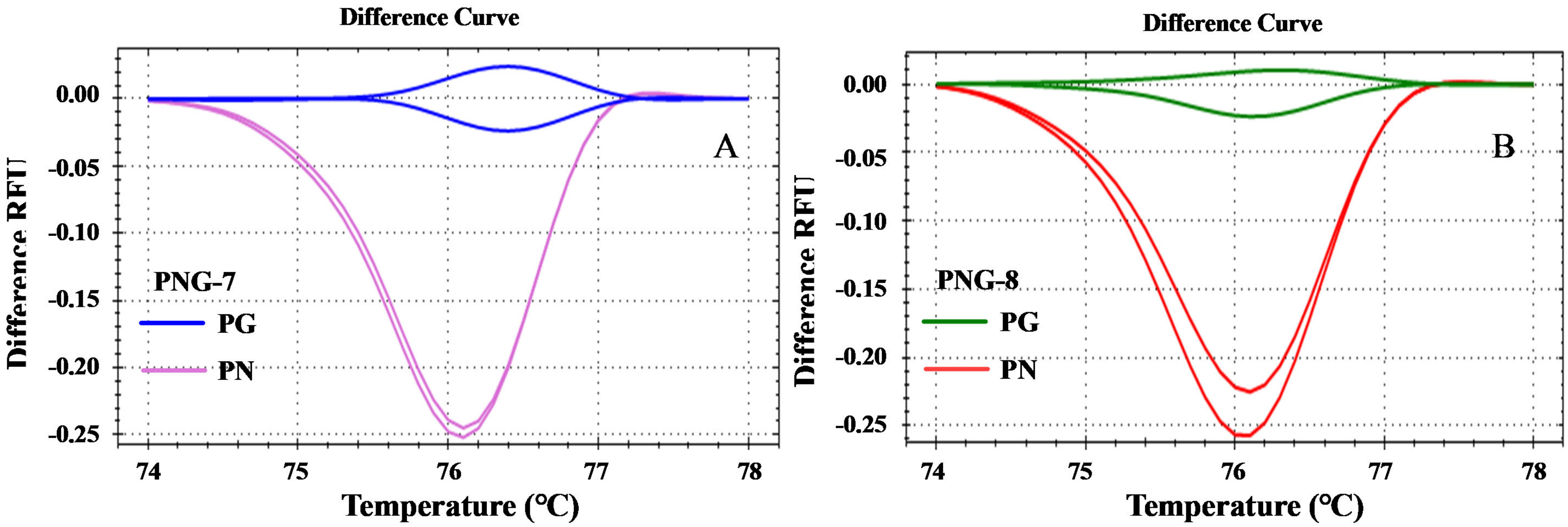

Figure S5. Difference curves analysis of flowers of *P. ginseng* (PG) and *P. notoginseng* (PN) using primers PNG-7 and PNG-8.

Table S4. Detailed in silico design parameters and specifications for the selected HRM primer pair PNG-2F/R

Parameter	Value / Sequence (5‘→3’)	Note
Forward Primer (PNG-2F)	AAGTTGCAAACCCATGGTCG	–
Reverse Primer (PNG-2R)	GTTGCCGAGAGTCGTTTGTG	–
Target Region	ITS2 (positions 92–253 in reference sequence OP001234.1)	–
Amplicon Length	162 bp (for both PG and PN)	Ideal for HRM analysis of potentially degraded DNA
Primer *Tm* (℃)	56.5 (PNG-2F), 59.3 (PNG-2R)	Difference (2.8 ℃) within optimal range
Primer GC Content	50.0% (PNG-2F), 55.0% (PNG-2R)	Within optimal range (40–60%)
Template Match	Perfect match to both PG and PN target sequences	Ensures high amplification efficiency
Predicted Amplicon *Tm* (℃)	~88.6 (PG), ~89.4 (PN)	Δ*Tm* ≈ 0.8 ℃, providing the basis for HRM differentiation
Software Score	676 (PG), 648 (PN)	High score indicates minimal secondary structure risk

Parameter	Value / Sequence (5‘→3’)	Note
Optimal Annealing Temp.	58.6 ℃ (software recommendation)	Experimentally validated and optimized

Note：Primer design was performed using Oligo 7 software. The objective was to design a single pair of universal primers capable of amplifying a homologous region containing diagnostic single nucleotide polymorphisms (SNPs) in both species, thereby ensuring identical amplicon length and primer binding efficiency for precise HRM comparison. The following constraints were applied during in silico design: amplicon length range of 80 – 250 bp, primer length of 18 – 22 nucleotides, primer melting temperature (*Tm*) between 55 – 65 ℃ with a maximum inter-primer *Tm* difference of 5 ℃, and GC content of 40 – 60%. Primer specificity was verified by in silico PCR against a local database containing Panax genus sequences. The primer pair PNG-2F/R, targeting the ITS2 region, met all criteria and was selected for subsequent validation.

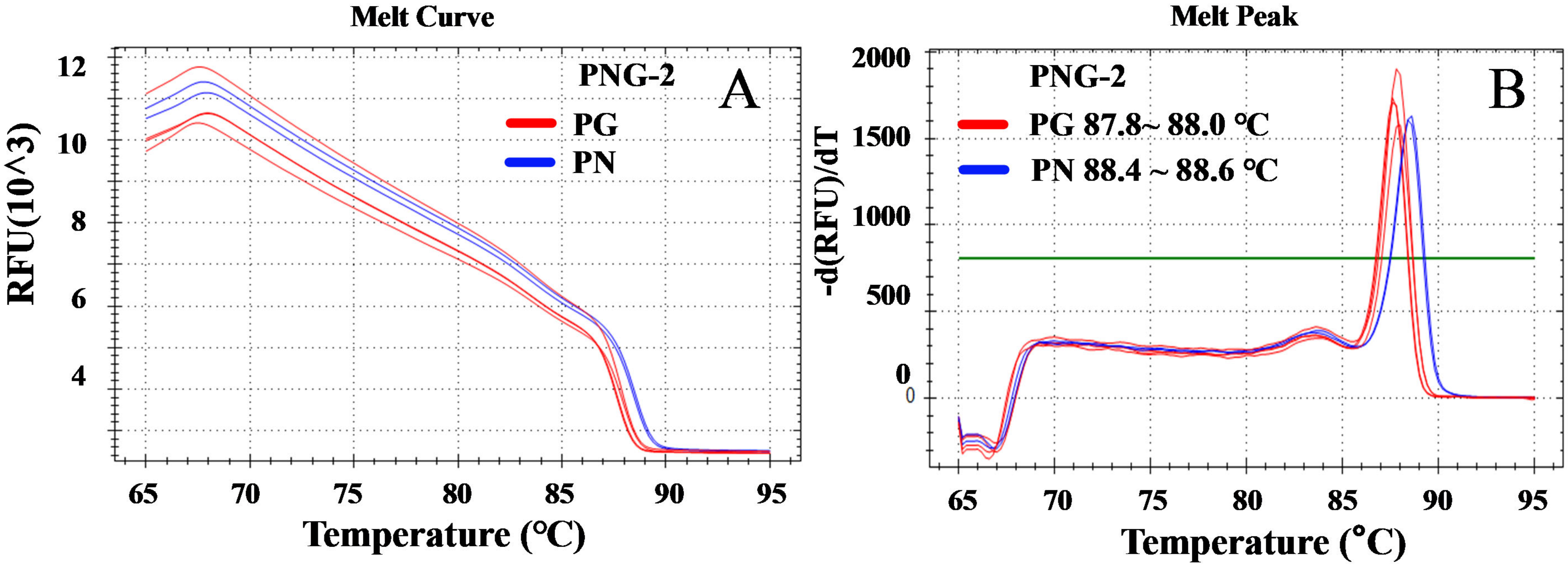

Figure S6. Rapid HRM screening results of *P. ginseng* (PG) and *P. notoginseng* (PN) processed products across different matrices.

Note: (A) Normalized melting curves and (B) derivative melting peaks (*Tm*) illustrate the diagnostic consistency of the PNG-2 assay. Red lines represent *P. ginseng* (PG) samples, including two batches of root slices and two batches of ultramicro powder (*Tm*: 87.8 – 88.0 ℃). Blue lines represent two batches of *P. notoginseng* (PN) root slices (*Tm*: 88.4 – 88.6 ℃). The high degree of spectral overlap confirms the method's robust matrix stability across diverse commercial processing forms.

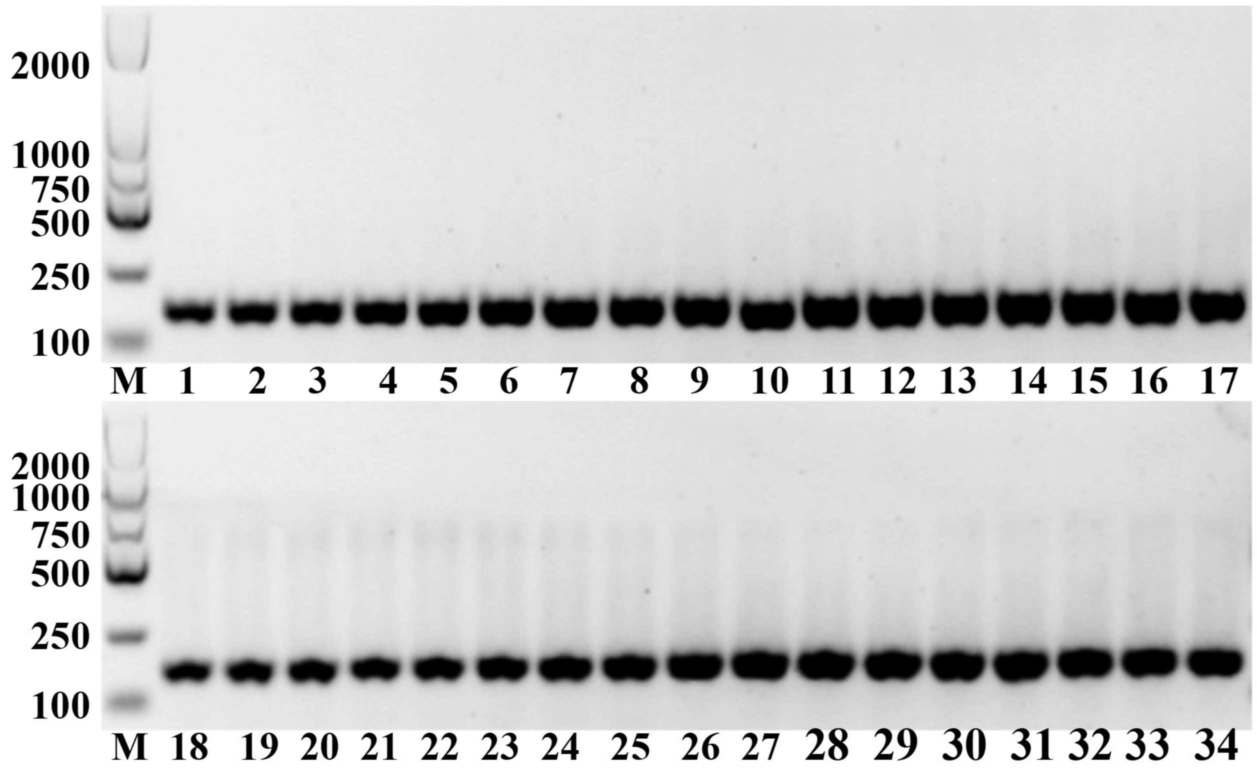

Figure S7. Agarose gel electrophoresis (1.5%) of the PCR products amplified with primer PNG-2 for the entire validation cohort.

Note: Lanes 1 – 26: *P. ginseng* (PG) samples (RS-01 – RS-26), comprising floral tissues, root slices, and ultramicro powders; Lanes 27 – 34: *P. notoginseng* (PN) samples (SQ-01 – SQ-08), including floral tissues and root slices. Lane M: DNA marker DL2000. The uniform presence of a specific target band at 162 bp across all 34 samples demonstrates the high amplification specificity and universality of the established primer set for the Panax genus.

Table S5. Sequence identity analysis of the PNG-2 amplicons from *P. ginseng* and *P. notoginseng*.

Region/Feature	Sequence (5' → 3')	Length (bp)	Identity (%)
Designed Target (PG)	AAGTTGCAAACCCATGGTCGGGGACC**A**CCCTTGGGTGG**AT**CTCGTCCGAACAACGAC<br>CCCCCGGCGCGGAATGCGCCAAGGAAATCAAA**C**TGAACTGCACGCGTCCCCCCCGTTT<br>GCGGGCGGCGGAAGCGTCTTTCTAAAACACAAACGACTCTCGGCAAC	162	N/A
Reference Sanger Seq PG, n = 22	AAGTTGCAAACCCATGGTCGGGGACC**A**CCCTTGGGTGG**AT**CTCGTCCGAACAACGAC<br>CCCCCGGCGCGGAATGCGCCAAGGAAATCAAA**C**TGAACTGCACGCGTCCCCCCCGTTT<br>GCGGGCGGCGGAAGCGTCTTTCTAAAACACAAACGACTCTCGGCAAC	162	100%
Designed Target (PN)	AAGTTGCAAACCCATGGTCGGGGACC**G**CCCTTGGGTGG**CC**TCGTCCGAACAACGACC<br>CCCCGGCGCGGAATGCGCCAAGGAAATCAAA**T**TGAACTGCACGCGTCCCCCCCGTTTG<br>CGGGCGGCGGAAGCGTCTTTCTAAAACACAAACGACTCTCGGCAAC	162	N/A
Reference Sanger Seq PN, n = 12	AAGTTGCAAACCCATGGTCGGGGACC**G**CCCTTGGGTGG**C**CTCGTCCGAACAACGACC<br>CCCCGGCGCGGAATGCGCCAAGGAAATCAAA**T**TGAACTGCACGCGTCCCCCCCGTTTG<br>CGGGCGGCGGAAGCGTCTTTCTAAAACACAAACGACTCTCGGCAAC	162	100%
**SNP Positions between PG and PN**	27 (A/G), 39 (A/C), 81 (T/C), 87 (C/T)		

This table illustrates the high degree of sequence conservation and the specific polymorphic sites between the amplified products of *P. ginseng* (PG) and *P. notoginseng* (PN). The sequences obtained from Sanger sequencing of four PG floral samples and three PN floral samples were 100% identical to the theoretical amplicons designed for the PNG-2 primer set. Four stable single nucleotide polymorphisms (SNPs) were identified within the 162 bp region, providing the thermodynamic basis for the distinct melting temperatures (*Tm*) observed in the HRM analysis. The consistency between the experimental sequencing data and the HRM results confirms the diagnostic precision of the developed assay.

Table S6. Comparison of advantages and disadvantages between DNA barcoding and HRM analysis.

Method	Advantages	Disadvantages
DNA barcoding	1. High species specificity;<br>2. Accurate and reliable identification;<br>3. Broad applicability;<br>4. Capable of identifying unknown species;<br>5. Data can be shared and cross-referenced	1. High cost and time-consuming (2–5 days per batch);<br>2. Requires sequencing equipment;<br>3. Relies on completeness of reference databases;<br>4. Data analysis is relatively complex;<br>5. High sample quality requirements
HRM analysis	1. Rapid and efficient (2–3 hours per batch);<br>2. Low cost；<br>3. Simple operation with closed-tube detection;<br>4. High throughput and high sensitivity;<br>5. Capable of detecting SNPs and mixed samples;<br>6. High reproducibility	1. Does not provide sequence information;<br>2. Requires prior sequence knowledge for primer design;<br>3. Depends on reference samples for comparison;<br>4. Sensitive to PCR conditions
DNA barcoding + HRM analysis	The integration of DNA barcoding and HRM analysis enables rapid, accurate, and cost-effective species identification, combining the reliability of sequencing with the efficiency of closed-tube high-throughput detection.	
